# Nanoarray-Embedded Hierarchical Surfaces for Highly Durable Dropwise Condensation

**DOI:** 10.34133/2022/9789657

**Published:** 2022-08-09

**Authors:** Yue Hu, Kaili Jiang, Kim Meow Liew, Lu-Wen Zhang

**Affiliations:** ^1^Department of Engineering Mechanics, School of Naval Architecture, Ocean and Civil Engineering, Shanghai Jiao Tong University, Shanghai 200240, China; ^2^State Key Laboratory of Low-Dimensional Quantum Physics, Department of Physics & Tsinghua-Foxconn Nanotechnology Research Center, Tsinghua University, Beijing 100084, China; ^3^Department of Architecture and Civil Engineering, City University of Hong Kong, Kowloon, Hong Kong SAR, China; ^4^Centre for Nature-Inspired Engineering, City University of Hong Kong, Kowloon, Hong Kong SAR, China

## Abstract

Durable dropwise condensation of saturated vapor is of significance for heat transfer and energy saving in extensive industrial applications. While numerous superhydrophobic surfaces can promote steam condensation, maintaining discrete microdroplets on surfaces without the formation of a flooded filmwise condensation at high subcooling remains challenging. Here, we report the development of carbon nanotube array-embedded hierarchical composite surfaces that enable ultra-durable dropwise condensation under a wide range of subcooling (Δ*T*_sub_ = 8 K–38 K), which outperforms existing nanowire surfaces. This performance stems from the combined strategies of the hydrophobic nanostructures that allow efficient surface renewal and the patterned hydrophilic micro frames that protect the nanostructures and also accelerate droplet nucleation. The synergistic effects of the composite design ensure sustained Cassie wetting mode and capillarity-governed droplet mobility (Bond number < 0.055) as well as the large specific volume of condensed droplets, which contributes to the enhanced condensation heat transfer. Our design provides a feasible alternative for efficiently transferring heat in a vapor environment with relatively high temperatures through the tunable multiscale morphology.

## 1. Introduction

Condensation is a ubiquitous phenomenon in nature and plays a key role across a wide range of industrial applications, such as anti-icing, power generation, sewage treatment, water desalination, and energy harvesting [1–3]. To enhance the condensation performances for higher heat transfer efficiency and further substantial savings in energy [1, 4], efficient nucleation of droplets [5] and rapid shedding [6, 7] are essential in surface designs. Considerable efforts have demonstrated that dropwise condensation, normally on hydrophobic surfaces, exhibits efficient heat transfer over filmwise condensation [3, 8]. Facilitated by lithography, etching, and the other straightforward approaches for surface modification, various anti-wetting designs are emerging, including pillars [9, 10], pit-array surfaces [11], multiscale surfaces [12–14], and hybrid surfaces [4, 15]. The customized and sophisticated designs enable control of the surface roughness, and optimization of the microtopographic parameters, thereby allowing regulation of the liquid-solid contact mode, further affecting the droplets dynamics, and manipulating the condensation performance [16].

However, sustaining droplet condensation at a high degree of subcooling (difference between the temperature of saturated vapor and the condensation surface [17]) remains challenging. Increasing subcooling will result in elevated nucleation density and condensation rate, which may exceed the droplet departure rate and form a liquid film [8, 18]. Therefore, the film-like droplets that pin on the surface will undergo the irreversible Cassie–Wenzel wetting transition, leading to the phenomenon of flooding that declines the heat transfer efficiency of the surface [19, 20]. In efforts to maintain the Cassie state of the droplet, previous studies have focused on decreasing the solid-liquid contact area [21, 22] or the number of droplet nucleation sites [23, 24] by designing increasingly smaller arrays or approximate arrays, from micro to nanoscale size, such as pine needle shapes [6], microconical architectures [25], nanoscaffolds [26], microratchet arrays [27], and vertical arrays of carbon nanotube (CNTs) [28, 29]. These surfaces indeed exhibit an enhancement in dropwise condensation by collecting a volume of water three times higher than that in the case of plate surfaces [30], achieving a 100% higher heat flux than that on the plane hydrophobic surface [31], and reaching a directional transport efficiency of approximately 80% for tiny droplets [27].

Nevertheless, two fundamental contradictions prevent the applications of micro/nanoarrays at large subcooling. The first one is the paradox of diminishing array size and mechanical robustness. Exposed array structures with high aspect ratios are considerably fragile and suffer from disruptions due to buckling, clustering, and fracture, even under a small external disturbance [32]; these disruptions eventually affect the wetting state and weaken the hydrophobic effect [33]. A recently armor-protected superhydrophobic surface presents the remarkably robustness and inspires the development of hierarchical surface [34]. The second one is the contradiction between high droplet mobility and accelerated nucleation. Hydrophobic array surfaces promise rapid droplet removal that is the key to dropwise condensation, but has a negative effect on droplet nucleation, especially at the initial stage of condensation [35, 36]. Conversely, hydrophilic surfaces promote the nucleation rate of droplets, but lead to undesired flooding.

In this study, we report an integrated design of a self-protected hierarchical hybrid surface that is composed of micropatterned surface structures and vertically arrayed carbon nanotubes (VACNTs) grown internally and directly. We propose that this approach is a complementary and enhanced strategy for achieving stable dropwise condensation efficiency at high subcooling. The robustness of our hierarchical patterned surfaces is demonstrated through a cyclic loading test. The CNT array, which is grown under control in restricted frames, is expected to act as the major hydrophobic component because of the nanoscale contact sites and the intrinsic water-repellency of CNTs. The hydrophilic micro-substrate patterned as a frame is expected to play a critical role in protecting the slender nanoarrays and providing essential hydrophilic sites that accelerate nucleation without compromising the global hydrophobicity of the surface. We perform a comprehensive analysis of the coalescence, jumping, and removal of droplets on the hierarchical surfaces. A heterogeneous nucleation model is developed for the nanoarray surface in the Cassie wetting state to reveal the underlying mechanism for dropwise condensation. Our experiments and molecular simulation are performed to show that the droplets and the density distribution of the water molecules are affected by the microtextured silicon (Si) substrate. This work enables a new microstructured surface with durable dropwise condensation and enhanced mechanical robustness at high subcooling under cyclic mechanical loading.

## 2. Results

### 2.1. Hierarchical CNT Array-Planted Patterned Surface

**Figure 1:**
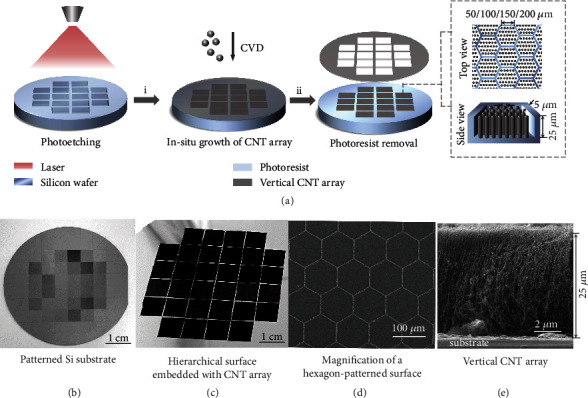
Features of superhydrophobic hierarchical VACNT-planted patterned surfaces. (a) Schematics showing the fabrication process for the hierarchical VACNT-planted patterned surfaces. (b) Patterned Si substrate. (c) Overall view the hierarchical surfaces embedded with CNT array in 1 cm × 1 cm pieces. (d) Higher magnification of a hexagon-patterned surface and (e) side view of the embedded VACNTs with a height of 25 *μ*m, as observed in scanning electron micrographs.

We designed and fabricated a hierarchical surface in which VACNTs are planted in a carefully patterned Si substrate (Figure 1). This bi-scale surface design involves two major steps. To construct the microscale structure, the Si wafer is patterned in triangular, circular, square, and hexagonal tessellation through photolithography. Unlike pillars, the interconnected indentations act as a stiff frame that plays the vital roles of protecting and limiting the deformation of inner nanostructures. Figure 1(a) shows the hexagonal pattern, with photolithographic cavities having a height of 25 *μ*m, an inter-cavity distance of 5 *μ*m, and cavity sizes of 50 *μ*m, 100 *μ*m, 150 *μ*m, and 200 *μ*m. The geometry and sizes of the triangular, circular, and square pits are shown in Figure S1. With regard to the nanostructure, we intend to plant vertical CNTs in the interconnected cavities by using chemical vapor-phase deposition (CVD), thus generating a hydrophobic surface with nanoscale contact sites. The efficient CVD approach for VACNTs has been explored in our previous studies [29, 37], and a detailed description is given in the methods. Undesired CNTs that grow on the walls of the microcavities can be eliminated by removing the photoresist mask, thus leaving only the VACNTs planted in the notches. This planting strategy ensures that the dense, slender nanostructures occupy most of the surface area and thus determine the global wettability of the surface. The contact sites between the intrinsically hydrophobic CNT arrays and the vapor offers reduced liquid-solid contact area, thereby promoting the mobility of droplets and increasing the condensation efficiency [38].

### 2.2. Dropwise Condensation on the Hierarchical Surface

**Figure 2:**
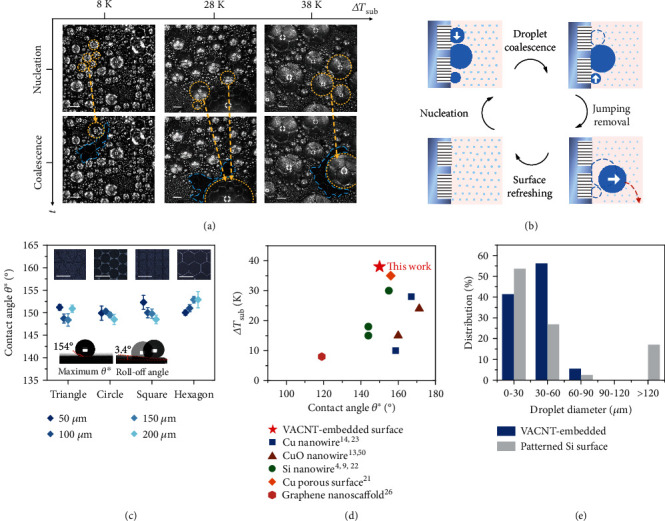
Condensation and properties of the hierarchical surface. (a) Optical records show nucleation and jumping coalescence on the hexagonal patterned surface with pattern size *L* = 150 *μ*m at three subcooling: ∆*T* = 8 K, ∆*T* = 28 K, and ∆*T* = 38 K. ∆*T*_sub_ is defined as the difference between the surface temperature and saturated vapor temperature. The yellow dashed circles represent the droplets before and after jumping coalescence, and the blue dashed lines represent the completely renewed surface after droplet coalescence. Scale bars = 200 *μ*m. (b) Schematic illustration of the condensation process. (c) The measured apparent contact angle (CA) *θ*^∗^ on surfaces with patterns of varied shapes and sizes. The inset images at the top are micrographs of the hierarchical surfaces with triangular, circular, square and hexagonal patterns. Scale bars = 150 *μ*m. The inset images at the bottom show the maximum value of *θ*^∗^ and the average value of the droplet roll-off angle. (d) Comparison of ∆*T*_sub_ achieved by the current hierarchical surface with that from the other nanowire or nanoarray surfaces. (e) Size distribution of the condensed droplet on the VACNT-Si surface and a pure-patterned Si surface of the same size, at ∆*T* = 28 K.

To understand the condensation dynamics on our hierarchical surfaces, we experimentally investigated the nucleation, growth, coalescence, and removal of the droplets under saturated water vapor.  Figure 2(a) confirms stable dropwise condensation on the hierarchical surface without film formation for a wide range of subcooling, up to a maximum of 38 K; the main reason for this observed behavior is that CNT arrays contribute to the surface superhydrophobicity. The wetting state measurements using optical microscopy show that the apparent CA, *θ*^∗^, reaches a maximum value of 154° and has an average value of 151°, with a roll-off angle of approximately 3.4° across a wide scenario (Figure 2(c)). As the Cassie state is maintained during the condensation process, the surface enables coalescence-induced jumping of neighboring droplets (yellow dashed lines in Figure 2(a)) on the condensed surface, subsequently exposing a completely renewed surface (blue dashed lines in Figure 2(a)) in the saturated vapor environment for a new cycle of condensation and heat exchange.  Figure 2(b) depicts an entire cycle, illustrating the nucleation, coalescence, jumping, and shedding of droplets on the hierarchical surface. The coalescence of nucleated droplets through jumping contributes to droplet mobility, resulting in a fast renewal of the surface and an increase in heat transfer efficiency [39]. From the thermodynamic perspective, after the droplets in the steam environment grow and coalesce, the surface energies of the *n* individual droplets (∑_*i*=1_^*n*^*E*_i_) on the coalescence path are converted to the surface energy of a merged droplet (*E*_2_); a certain amount of this energy is released (Δ*E*_s_ = ∑_*i*=1_^*n*^*E*_i_ − *E*_2_), which provides the kinetic energy for the jumping of droplets and drives coalescence.

Noted that in comparing to Si nanowire, copper nanowire, and other nanoarray surfaces that undergo a transition from dropwise to filmwise condensation at an averaged surface subcooling 20 K in subcooling tolerance, our hierarchical surfaces achieve an improvement of around 18 K in subcooling tolerance (Figure 2(d)). The dropwise condensation process at Δ*T*_sub_ =8 K−38 K is shown in Videos S1, S2, and S3. In all three cases, droplets move and depart by the coalescence-induced jumping rather than sliding, and the probability of droplet departure decreases with the increasing Δ*T*_sub_. This is due to the high heat flux caused by the large Δ*T*_sub_ form uncontrolled nucleation in some microgaps between CNTs, which provides large adhesive force on droplets [31]. To explain the maintenance of dropwise condensation at high Δ*T*_sub_, we introduce the dimensionless Bond number (*Bo*) to determine the dominant driving force in the movement of a droplet. Defined in terms of the ratio between the gravitational and capillary forces, *Bo* is given by *ρgR*_*f*_^2^/*γ*_LG_ (where *ρ* is the condensate density, *g* is the gravitational constant, *R*_f_ is the droplet radius just before the jumping movement, and *γ*_LG_ is the surface tension), indicating capillarity-controlled (Bo < 1) or gravity-controlled droplet mobility (*Bo* > 1), corresponding to the desired dropwise and undesired filmwise condensation, respectively [36, 40]. In our experimental measurements, the maximum radius of the jumping droplets, *R*_f_, ranges from approximately 164 *μ*m (Δ*T*_sub_ = 8 K) to approximately 613 *μ*m (Δ*T*_sub_ = 38 K), and *γ*_LG_ falls within the range of 72.8 mN/m–67.1 mN/m as the temperature elevates from 293 K to 328 K [41], yielding a maximum value of *Bo* in the range 0.004–0.055. Note that the predicted result for *Bo* ≪ 1 is within the well-known domain of capillary-governed droplet departure and dropwise condensation [36, 40]. The removal of the capillarity-dominated droplets relies on not only the gravity, but also the coalescence-induced jumping [42]. Therefore, the small *Bo* number reveals the fundamental reasons for the maintenance of the spherical droplet shape and the efficiency of surface renewal at high subcooling.

To further identify the advantages of the hierarchical surface during dropwise condensation, we studied the formation of discrete droplets and compared it with the corresponding scenario for a micropatterned substrate without implanted nanoarrays. It is worth noting that the Si surface is intrinsically hydrophilic (*θ*^∗^ ≈ 75°), and even though *θ*^∗^ reaches 121° at the room temperature of 24 °C (indicating hydrophobicity) after lithography (cavities with a feature size of 150 *μ*m) (Figure S2(a)), the formation of irregular films or puddles on the condensing patterned Si substrate is in sharp contrast to the dropwise condensation on CNT-implanted surfaces (Figures S2(b) and S2(c)). This discrepancy is manifested in the distribution of the diameters of discrete droplets. As evidenced in Figure 2(e), in the case of the hierarchical surfaces, 95% of the droplets have a diameter less than 60 *μ*m, whereas in the case of the pure patterned Si surface, a major portion of the surface is occupied by large irregular droplets (17% of the droplets have an equivalent diameter *D* ≥ 120*μ*m) in the absence of nanoarrays. As the hierarchical surfaces have condensed droplets of smaller size with much higher *θ*^∗^, it plays a substantial role in declining the thermal resistance between vapor and the substrate during condensation, especially at high subcooling [14]. As another essential indicator of surface condensation, the thermal diffusivity coefficient of the solid substrate is quantified in Figure S3. It increases from 38 mm^2^/s for a patterned Si surface to a maximum value of 87 mm^2^/s and an average value of 75 mm^2^/s for a hierarchical surface. This substantial increase of 97% in the thermal diffusivity indicates that the CNT-implanted surfaces exhibit better heat conduction performance, which endows the hierarchical surfaces with an enhanced condensation property.

### 2.3. Heat Transfer Performance Dominated by the Nanoscale Arrays

**Figure 3:**
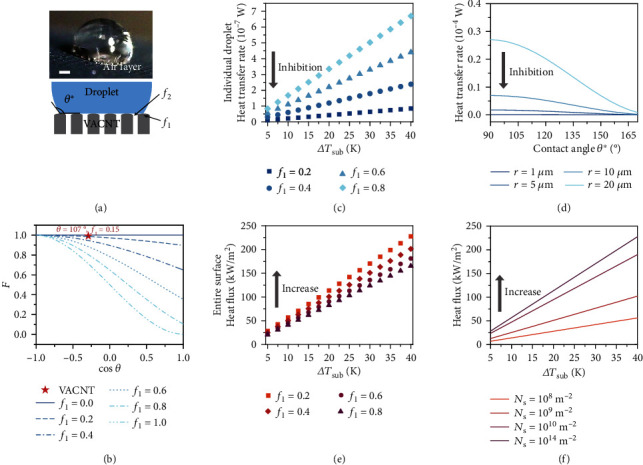
Heat transfer properties for condensation on the nanoarray structure in the Cassie wetting mode. (a) Optical image of the solid-liquid air layer and schematic diagram of droplet in the Cassie wetting state for VACNT with a nanoarray structure. Scale bar = 500 *μ*m. (b) The Fletcher factor *F* varies with the roughness factor *f*_1_ and the intrinsic contact angle *θ* of surfaces. Condensation heat transfer rate for an individual droplet as a function of (c) ∆*T*_sub_ for different values of *f*_1_ (*θ*^∗^ = 150°, *r* = 1 *μ*m) and (d) the apparent contact angle *θ*^∗^ for different values of droplet radius *r* (∆*T*_sub_ = 5 K and *f*_1_ = 0.5). Condensation heat flux of the entire surface as a function of (e) ∆*T*_sub_ for different values of *f*_1_ (*θ*^∗^ = 150°, *r* = 1 *μ*m, and *N*_s_ = 10^14^ m^−2^) and (f) ∆*T*_sub_ for different values of nucleation density *N*_s_ (*θ*^∗^ = 150°, *r* = 1 *μ*m, and *f*_1_ = 0.2).

To gain insights into the physical mechanisms underlying stable dropwise condensation, we laid emphasis on the significant contribution of nanoscale vertical arrays in enabling efficient heat transfer.  Figure 3(a) shows air pockets at the solid-liquid interface, demonstrating that the CNT array maintains the Cassie wetting state, as remarked in previous studies [30, 43, 44]. Furthermore, as seen in Figure 2(a), the Si microframes do not have a pinning effect on the three-phase contact line of droplets, which remains circular rather than transforming into the shape of the pattern. These measurements evidence the role of nanoarrays in determining the wetting state and condensation efficiency at high subcooling.

Previous studies have proven that the surface properties have an obvious influence on the nucleation rate [8, 16, 45]. However, the description for vapor nucleation on the array-like surface is inadequate in terms of the surface heterogeneity. Here, we developed a heterogeneous nucleation model for a nanoarray surface in the Cassie wetting state and specifically investigated the contribution of the nanoarray on the heat transfer performance. As the Gibbs free energy Δ*G*_het_ for heterogeneous nucleation from classical nucleation theory is restricted to ideal smooth surfaces [46], we modified it by splitting the term for surface free energy into a term related to the spherical cap and a term related to the bottom contact area, while keeping the volume term unchanged:

(1) $$\begin{array}{c} \Delta G_{\text{het}}=\gamma_{\text{LG}}S+\left[\left(\gamma_{\text{SL}}-\gamma_{\text{SG}}\right)f_{1}+\gamma_{\text{LG}}f_{2}\right]A+\Delta g_{v}V, \end{array}$$

where *S* = 2*πR*^2^(1 − cos*θ*^∗^) is the outer surface area of the spherical cap of the droplet with radius *R*, *A* = *πR*^2^(1 − cos^2^*θ*^∗^) is the bottom area of the droplet cap, and *V* = *πR*^3^(1 − cos*θ*^∗^)^2^(2 + cos*θ*^∗^)/3 represents the volume of the droplet with an apparent CA *θ*^∗^. *γ*_LG_, *γ*_SL_, and *γ*_SG_ represent the liquid-gas, solid-liquid, and solid-gas surface tension, respectively. *f*_1_ is the solid-liquid contact area fraction, and *f*_2_ = 1 − *f*_1_ is the liquid-gas contact area fraction. Δ*g*_*v*_ is the difference in free energy per unit volume for water and supersaturated vapor at the same pressure. Subsequently, the heterogeneous nucleation energy barrier or the work of critical nucleation Δ*G*_het_^∗^ can be derived by using *∂*Δ*G*_het_/*∂R* = 0, which gives

(2) $$\begin{array}{c} \Delta G_{\text{het}}^{*}=\frac{4\pi }{3\Delta g_{v}^{2}\left(1-\cos\theta^{*}\right){\left(\cos\theta^{*}+2\right)}^{2}}{\left[\left(3+\cos\theta^{*}\right)\gamma_{\text{LG}}+f_{1}L\left(\cos\theta^{*}+1\right)\right]}^{3}, \end{array}$$

where *L* = *γ*_SL_ − *γ*_SG_ − *γ*_LG_.*θ*^∗^ satisfies cos*θ*^∗^ = *f*_1_(1 + cos*θ*) − 1 based on Cassie theory, and cos*θ* = (*γ*_SG_ − *γ*_SL_)/*γ*_LG_ according to Young's equation, where *θ* is the intrinsic CA. Drawing from the expression for homogeneous critical nucleation energy Δ*G*_hom_^∗^ from classical nucleation theory, Δ*G*_het_^∗^ can be reformulated as

(3) $$\begin{array}{c} \Delta G_{\text{het}}^{*}=F\frac{16\pi \gamma_{\text{LG}}^{3}}{3\Delta g_{v}^{2}}=F\Delta G_{\mathrm{hom}}^{*}. \end{array}$$

*F* = *f*(*θ*, *f*_1_) = [2 − *f*_1_(1 + cos*θ*)]^2^[1 + *f*_1_(1 + cos*θ*)]/4 is a critical scaling factor residing in the range [0, 1], termed as Fletcher factor. Although another form of *F* was derived in a previous study for describing fractal surface [47], here it quantitatively characterizes the dependence of the surface geometry and the intrinsic wetting property on heterogeneous nucleation. A larger value of *F*, i.e., closer to 1, implies that a higher energy barrier must be overcome to form condensed droplets and also suggests that critical heterogeneous nucleation tends to be extremely close to ideal homogeneous nucleation.

By leveraging the proposed model, Figure 3(b) elaborates the relationship between the Fletcher factor *F* and the two critical characteristic parameters of a surface: the intrinsic wetting angle *θ* and the solid-liquid contact area fraction *f*_1_, which can be related to the density of nucleation sites in the Cassie state. The predictions indicate that surfaces with a larger *θ* or a smaller *f*_1_ tend to suppress droplet nucleation. As CNT array surfaces have a *θ* of approximately 107° [48] and a small *f*_1_, which is identified to be 0.15 when measured using the image contrast approach (Figure S4), *F* is extremely close to 1.0, as shown in Figure 3(b) (represented by a red star). The large work-of-critical-nucleation value implied by the high *F* indicates that it is difficult to pin droplets on the nanoscale arrayed surface, leading to favorable conditions for droplet mobility, surface renewal, and maintenance of dropwise condensation.

To quantify the effect of surface roughness on the condensation performance at altered subcooling, we studied the heat transfer efficiency and heat flux, which are predominantly influenced by the CNT array surfaces (Figures 3(c)–3(f)). The heat transfer models for an individual droplet and a group of droplets are used to characterize the heat transfer rate and heat flux (Supplementary Section S5). As illustrated in Figure 3(c), the heat transfer rate of a single droplet on a rough surface with *f*_1_ = 0.2 (relatively small solid-liquid contact area) shows a decrease of 87% when compared with the corresponding value for a smooth surface with *f*_1_ = 0.8 (relatively large solid-liquid contact area) at a high subcooling of 40 K. This decrease in the case of a rough surface is attributable to the air layer trapped in the cavities, which has a lower thermal conductivity than a smooth surface. With regard to the influence of the apparent CA *θ*^∗^ and the droplet radius *r*, Figure 3(d) suggests that the condensation of a small droplet decreases the heat transfer rate to a greater extent than does the condensation of a large droplet and this decrease can be up to 1 order of magnitude for *θ*^∗^ =90°. For condensed droplets with the same radius, a large value of *θ*^∗^ minimizes the heat transfer rate for individual droplets; therefore, the heat-sensitive surface tension of the droplet is maintained, preventing flooding of the surface.

The global heat transfer efficiency of the surface was further investigated with respect to variable *f*_1_. In Figure 3(e), the heat flux shows a maximum increase of 38%, when *f*_1_ decreases from 0.8 to 0.2 at Δ*T*_sub_ =40 K. Furthermore, the surface with a smaller value of *f*_1_ facilitates the condensation of a larger number of droplets with radius less than 100 *μ*m (Figure S5(b)), and the increased nucleation density *N*, which denotes the number of nucleation sites per unit area on the condensing surface, increases the heat flux across the surface, as shown in Figure 3(f). Therefore, the nanoscale array structures raise the energy barrier for droplets nucleation and optimize surface heat transfer performance.

### 2.4. Condensation Activation Governed by the Hydrophilic Microstructures

**Figure 4:**
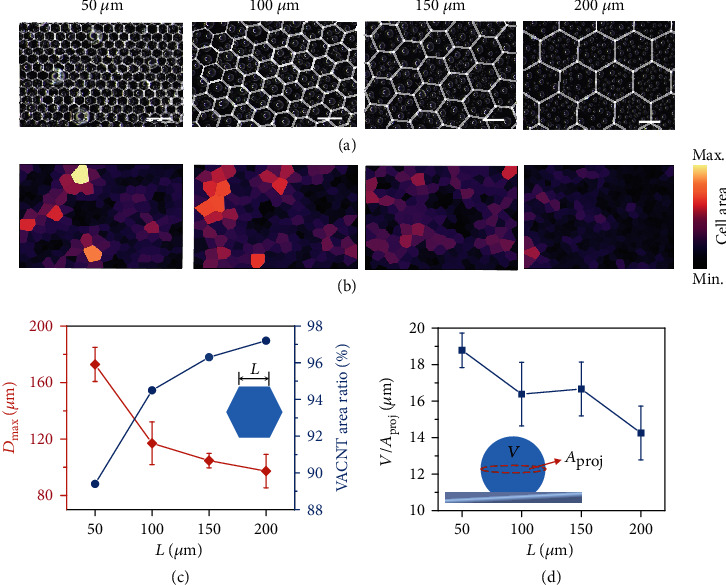
Nucleation activation on the hierarchical surfaces with different pattern sizes for ∆*T*_*sub*_ = 28 *K*. (a) Optical images of the condensation performance on the hierarchical surfaces as the pattern size *L* increases from 50 *μ*m, 100 *μ*m, 150 *μ*m to 200 *μ*m. White lines in images delineate the edges of the pattern on hierarchical surfaces. The corresponding surface fractions of Si are 10.6%, 5.5%, 3.7%, and 2.8%. Scale bars = 200 *μ*m. (b) Corresponding Voronoi representations of droplet distribution maps, colored according to the cell coverage area. (c) Maximum diameters of condensed droplets and proportion of VACNT area on different surfaces as a function of surface pattern size *L*. (d) Ratio between droplet condensation volume and projection area *V*/*A*_proj_ on the hierarchical surfaces for varying *L*.

Although the uncovered narrow wall of the microframes occupies a maximum area of only 10% of the surface in our experiments (*L* = 50 *μ*m), the hydrophilic nature of Si is likely to have an advantageous effect on condensation efficiency. To determine the potential influence of this patterned Si surface occupying a small percentage of the area, we investigated the distribution of condensed droplets of different sizes as the tessellation length *L* shortens from 200 *μ*m to 50 *μ*m, and the corresponding hydrophilic contact area enlarges from 3% to 10% (Figure 4).

**Figure 5:**
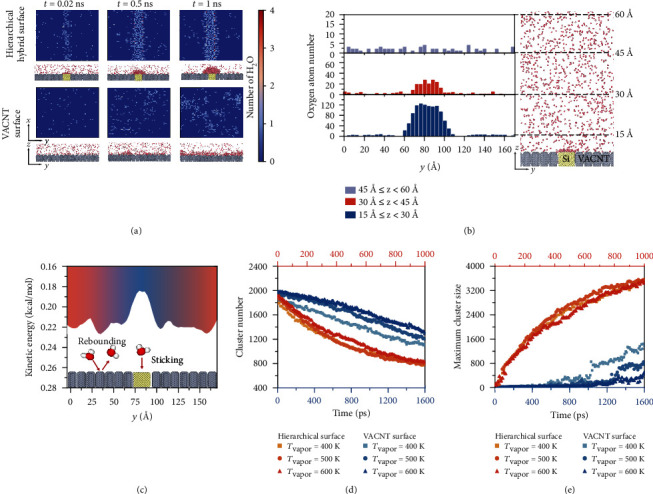
Comparison of the behavior of water molecules during condensation on the VACNT-Si hierarchical surface and the VACNT surface. (a) Images of the water molecule density map during the condensation process on the heterogeneous VACNT-Si surface and the VACNT surface. The inset images are corresponding snapshots of the molecular dynamics simulation near the surface. (b) Number of oxygen atoms at different distances (in the range 15 Å–60 Å) from the surface. (c) Variation in kinetic energy of water molecules along the *y*-axis. (d) Number of clusters and (e) maximum cluster size as a function of simulation time on the VACNT-Si hierarchical surface and the VACNT surface. The temperature of wall is set to 300 K, while the temperature of vapor is set to 400 K, 500 K, and 600 K to simulate various temperature difference between vapor and condensing substrate. A cluster of water molecules is determined by an intermolecular distance ≤ 3.36 Å.

At the very initial stage of condensation (10 s), microdroplets nucleate on the hydrophilic silicon frame, in the width of only 5 *μ*m (Figure S6). The surface with *L* = 50 *μ*m displays the largest coverage ratio of condensed droplets than others (Figure S7). After 30 s of condensation, as displayed in Figure 4(a), the size of the droplets increases considerably, and the nucleation density decreases, along with an increase in *L* and a growing area exhibiting local hydrophilicity (original images shown in Figure S8). From the corresponding Voronoi analysis of droplet distribution, the local areal proportions around large droplets are apparently higher than those around small droplets (Figure 4(b)). As the local areal proportion is governed by the distance between adjacent droplets, the results suggest that a larger area of the heat transfer surface is exposed to the vapor environment as a greater number of large droplets condense on the hybrid hierarchical surface. Figure 4(c) shows additional statistics on the maximum diameter of condensed droplets, which decreases from 172 ± 12 *μ*m at *L* = 50 *μ*m to 97 ± 11 *μ*m at L = 200 *μ*m, indicating that the densely patterned surface favors the collection of droplets in large volumes (the entire size distribution is shown in Figure S9). However, this outcome gives rise to another issue—large condensed droplets result in a large coverage area that hinders heat transfer between the surface and vapor. To extract the dominant factor in nucleation efficiency at the initial stage of condensation, the trend for the specific volume, defined by *V*/*A*_proj_, against pattern density is obtained, as illustrated in Figure 5(d), where *V* denotes the spherical crown volume of the droplets, *A*_proj_ is the maximum projection area, and the average value of *θ*^∗^ is 151°, from the previous characterization. The results indicate that for the hierarchical surface, the specific volume when *L* = 50 *μ*m is 32% higher than the specific volume when *L* = 200 *μ*m. Although a large drop volume and a large coverage area constitute a paradox for condensation efficiency, the large-sized droplets favored by hydrophilic sites exhibit a balance between the volume of condensed droplets and the corresponding coverage area (Figure S10); thus, the hierarchical surface with a highly dense pattern favors condensation of highly efficient condensation without affecting the heat transfer performance.

To provide insights into the influence of hydrophilic microframes on condensation behaviors at the molecular level, we carried out a comparative study of a hybrid surface and a hydrophobic VACNT surface through molecular dynamics simulation (models are shown in Figure S11). The density map images show that fast nucleation of water molecules occurs in the hydrophilic domain, whereas numerous droplets tend to scatter on the VACNT (Figure 5(a)). From the kinetic energy distribution of H_2_O near the hybrid surface (Figure 5(c)), it can be noted that the hydrophilic Si region promotes strong interaction between vapor and the substrate and exhibits apparently low kinetic energy, whereas the CNTs provide a large amount of kinetic energy for motion of H_2_O, leading to the rebounding of water molecules and the prevention of nucleation [49]. Furthermore, the hydrophilic nucleation site affects the density distribution of water molecules away from the surface, which is observed in the statistics related to the number of water molecules per vapor bin (Figure 5(b)). This behavior is attributable to the equilibrium process of evaporation and condensation for the nucleated droplets, which promotes the collection of a great number of adjacent water molecules near the surface and leads to apparent aggregation in the subsurface (15–45 Å). The increased cluster size and the rate of condensation nucleation on the hierarchical surface show that the hydrophilic microstructure provides localized activation sites that accelerate the nucleation and growth of droplets (Figures 5(d) and 5(e)). The difference between the vapor and the wall temperature increases the number of nucleation cluster on the VACNT surface with smaller cluster size, but is not apparent on the hierarchical surface. Thus, the hierarchical hybrid design that integrates hydrophilic microframes with hydrophobic CNT arrays promotes the rate of droplet nucleation and improves condensation efficiency while maintaining both surface hydrophobicity and droplet mobility.

### 2.5. Mechanical Robustness of the Hierarchical Surface

**Figure 6:**
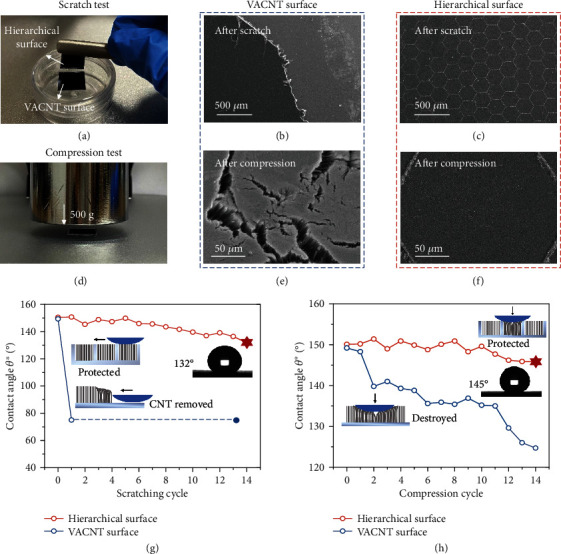
Robust stability of the hierarchical patterned surface. (a) Scratch test conducted using a steel scraper as the tangential load instrument on the hierarchical surface and the VACNT surface. SEM images of (b) the VACNT surface and (c) the hierarchical surface after scratching. (d) Compression setup, including the surface placed between the 500 g weight and the operating desktop. SEM images of (e) the VACNT surface and (f) the hierarchical surface after compression. Evolutions of the averaged apparent contact angle *θ*^∗^ from three independent measurements after (g) the cyclic scratch test and (h) the cyclic compression test and schematic diagrams (inset) of the protection and destruction mechanisms of surfaces during the cyclic robust tests.

Unlike fragile array structures featuring a large ratio between length and diameter, our hierarchical material enhances structural robustness when VACNTs are planted inside the interconnected cavities. We conducted cyclic scratching and compression tests to examine the stability of the designed hierarchical surfaces in the wetting state. Under cyclic scratching, the nanoarrays remain in the cavities with only slight damage to the hierarchical surfaces (Figure 6(c)); in contrast, on the substrate that is not protected by stiff frames, no trace of the CNTs remains (Figure 6(b)). Under cyclic compressive loading, cracks appear on the array surface (Figure 6(e)); however, the patterned hierarchical surface remains intact, without noticeable damage (Figure 6(f)). To test the robustness of the wetting property, the average CAs were measured after each scratch and compression load during cyclic testing. As shown in Figure 6(g), *θ*^∗^ of the hierarchical surface remains greater than 130° after 13 rounds of cyclic scratching, whereas the bare VACNT surface loses hydrophobicity. After the compression cycle, *θ*^∗^ of the hierarchical surface remains greater than 145°, with a maximum decrease in value of 2.8%, which is superior to the unprotected array, whose hydrophobicity weakens significantly (to approximately 125°) (Figure 6(h)). Thus, the interconnected micro-indentations offer a stiff frame for the fragile CNTs, ensuring mechanical robustness of the hierarchical structure under interruptions and preventing a wetting state transition.

## 3. Discussion

In this study, we fully exploit the advantages of distinct scales of array structures with different wetting characteristics, creating a superhydrophobic hierarchical patterned surface by integrating hydrophobic nanoscale vertical CNT arrays and hydrophilic microscale frames. The results show that the surface exhibits high endurance in a wide range of subcooling, 8 K–38 K, and exhibits highly stable dropwise condensation with good condensation efficiency; the surface outperforms conventional hydrophobic nanoarray/microarray surfaces that switch to flooding at an average Δ*T*_sub_ of 20 K [4, 9, 13, 14, 21–23, 26, 50]. Our experimental and theoretical study reveals that the high-endurance dropwise condensation is mainly attributable to the CNT arrays, which improve the surface properties in terms of high water repellency, better thermal performance, and reduced solid-liquid contact area owing to the nanoscale contact area fraction. Previous studies have shown the benefits of using CNT arrays in steam generation, evaporation-assisted detection, and dew harvesting [30, 51, 52], while in this study, we demonstrate the superior potential of CNT arrays for efficient condensation in extreme environments.

Through further exploring the effects of CNT arrays on critical nucleation and heat transfer, our surface with implanted CNT arrays shows a substantial enhancement of 97% in the thermal diffusivity coefficient when compared with a micropatterned Si surface without implanted CNT arrays. Furthermore, we find that the hydrophilic substrates patterned as microframes accelerate droplet nucleation by providing active nucleation sites for saturated vapor without compromising on the hydrophobicity of the entire surface. The specific volume of droplets increases by 35%, indicating a larger droplet size for the same coverage area and resulting in higher condensation efficiency, which is determined through kinetic energy analysis from molecular simulation. The interconnected microframes promise regionalization and confinement of the planted CNT array under mechanical perturbation, thereby conferring structural stability to of the surface. Thus, the integrated design results in increased droplet mobility and efficient droplet nucleation, which speeds up surface renewal; consequently, the condensation efficiency at high subcooling improves. Based on the current design, the VACNT-embedded hierarchical surfaces can be extended by different etching processes on various substrate materials to fulfill requirements for not only advanced condensation but also considerable use in anti-fogging, anti-icing, and highly efficient heat transfer applications.

## 4. Materials and Methods

### 4.1. Sample Preparation

Commercial 4-inch Si wafers (Suzhou Research Materials Microtech Co., Ltd., China) with a thickness of 0.5 mm were first patterned with microstructures by using photolithography; then, patterned cavities with a depth of 25 *μ*m were fabricated using dry etching. Next, the wafers were deposited with 5 nm Fe as catalyst by using an e-beam evaporator, after which a lift-off process was carried out to remove the photoresist. Finally, the microfabricated Si wafer was placed in a low-pressure CVD system to grow VACNTs by using acetylene as precursor. The reaction took place at a temperature of 973 K. Multiwalled CNTs with a diameter of 15 nm ± 5 nm were grown for 4 min. The surface samples were cut into pieces of 1 cm × 1 cm for subsequent testing. A field-emission scanning electron microscope (SEM, FEI, Sirion 200) was used to characterize the surface morphology and the lateral profile of the VACNTs in the cavities.

### 4.2. Condensation Experiments

Condensation tests were conducted under standard atmospheric pressure. Two types of steam conditions are prepared: ambient atmospheric conditions (~297 K) and hot steam at ~327 K. The hierarchical surfaces of different sizes were vertically placed on a cold source platform to attain temperatures of ~299 K and~289 K in different cases. Results from tests at three values of Δ*T*_sub_ were analyzed: the cooling surface under ambient temperature conditions (Δ*T*_sub_= 8 K), the room-temperature surface in water-vapor steam (Δ*T*_sub_= 28 K), and the cooling surface in water-vapor steam (Δ*T*_sub_= 38 K). The condensation processes were visualized and recorded under an optical microscope. Statistics related to droplet distribution and size were obtained by frame analysis methods using the Nano Measurer 1.2 software package.

### 4.3. Contact Angle (CA) Test

The apparent CAs were measured using a CA meter (KRUSS, DAS 100). The CA values were tested using a droplet volume of 10 *μ*L, and the average of the values at three positions on each sample was computed.

### 4.4. Heat Conduction Measurement

The heat transfer diffusivity coefficients of the surfaces were measured at room temperature by using a laser thermal conductivity meter (NETZSCH, LFA 467 HyperFlash). The surface heating process was recorded, and the thermal diffusivity of the sample at room temperature was given by

(4) $$\begin{array}{c} \alpha =0.1388\times \frac{d^{2}}{t_{50}}, \end{array}$$

where *d* represents the thickness of the surface and *t*_50_ is the time required to reach half of the peak value of temperature.

### 4.5. Mechanical Tests

Scratching tests were conducted using a steel scraper that provided tangential force, and compression tests were carried out using a 500 g weight loaded vertically on the surfaces. After each robustness test, the CA values were measured three times, and the average value was calculated. Each testing cycle was conducted 15 times.

### 4.6. Molecular Dynamics (MD) Simulations

In the condensation simulations, a uniform VACNT surface (modeling the CNT area in cavities) and a heterogeneous surface (modeling the hydrophilic edges near hydrophobic zones) were studied and compared. A periodic boundary was applied to the simulation box with the size of 151 Å × 176 Å × 250 Å. In the two surface models, the CNTs arranged in an orderly manner had a height of 15 Å, and the heterogeneous surface model had a hydrophilic zone in the middle. The hydrophilic Si region was modeled as a diamond lattice structure with a lattice constant of 5.43 Å.

For the solid surface, the carbon-carbon atom interaction was described using the AIREBO potential [53], whereas the Si-Si and Si-carbon interactions were described using Tersoff potential energy [54]. For the water droplets, the SPC/E rigid water model [55] was used; in this model, the water molecules had an internal bond angle of 109.47° and a bond length of 1 Å. Coulomb long-range interactions were applied to both hydrogen and oxygen atoms for producing, and a Lennard-Jones (LJ) potential was applied only to oxygen atoms for generating van der Waals interactions. The LJ potential was also used to describe the interaction between water molecules and solid surfaces. The LJ potential parameters were set as follows: *σ* = 3.41 Å and *ε* = 1.46 kJ/mol for Si–O interaction, and *σ* = 3.19 Å and *ε* = 0.31 kJ/mol for C–O interaction. The LJ potential and electrostatic terms were both cut off at 15 Å using the long-range solver of the particle-particle particle-mesh solver (PPPM) summation method. All simulations were carried out using the LAMMPS software [56].

A time step of 2 fs was used for all simulation processes. The solid surface atoms at the bottom of 3 Å were fixed, with all forces on atoms set to zero during the entire simulation. At the start, the vapor region consisting of 2093 water molecules was heated to 500 K in a canonical ensemble (NVT), and the unfixed surface atoms were relaxed at 300 K for 60 ps. Then, the vapor molecules were gradually cooled by heat transfer from the cold source surface in a microcanonical ensemble (NVE), while the surface remained under NVT thermodynamic control at 300 K. The duration of the process was 1 ns, and nuclei were formed on the surface. A cluster of H_2_O nuclei is defined as a set of H_2_O molecules, each of which is located at a distance of less than 3.36 Å from at least one other molecule in the cluster.

## Data Availability

All relevant data that support the findings are available within this article and supporting information and are also available from authors upon reasonable request.

## Supplementary Materials

Supplementary 1 Section S1. Patterned surfaces with different shapes. Section S2. Transition from filmwise to dropwise condensation after planting of VACNTs. Section S3. Thermal diffusivity of the hierarchical surfaces. Section S4. Contact area fraction measurement on the VACNTs. Section S5. Condensation model for heat transfer rate. Section S6. Initial stage of condensation on the hierarchical surfaces. Section S7. Size distribution of condensation droplets on different surfaces. Section S8. Size-dependent analyses of condensation efficiency. Section S9. Snapshots of condensation process on the heterogeneous surface and on the as-grown VACNT surface.

Supplementary 2 Video S1. Condensation on the hierarchical surface at a subcooling of 8 K.

Supplementary 3 Video S2. Condensation on the hierarchical surface at a subcooling of 28 K.

Supplementary 4 Video S3. Condensation on the hierarchical surface at a subcooling of 38 K.
