# Supplementary material for: Nanoarray-Embedded Hierarchical Surfaces for Highly Durable Dropwise Condensation
Source: Research (Wash D C). 2022 Aug 9;2022:9789657. doi: 10.34133/2022/9789657 (PMC9394060; doi:10.34133/2022/9789657)
Supplement: Supplementary 1 — Section S1. Patterned surfaces with different shapes. Section S2. Transition from filmwise to dropwise condensation after planting of VACNTs. Section S3. Thermal diffusivity of the hierarchical surfaces. Section S4. Contact area fraction measurement on the VACNTs. Section S5. Condensation model for heat transfer rate. Section S6. Initial stage of condensation on the hierarchical surfaces. Section S7. Size distribution of condensation droplets on different surfaces. Section S8. Size-dependent analyses of condensation efficiency. Section S9. Snapshots of condensation process on the heterogeneous surface and on the as-grown VACNT surface. [file 9789657.f1.docx]

**S1. Patterned surfaces of different shapes**

We design and fabricate the patterned surfaces of different shapes (hexagon, triangular, circular, and square) and edge sizes (50, 100, 150, and 200 μm). Variable surfaces were integrated on one silicon wafer to ensure their consistent environment for growing VACNT. The patterned wafers were divided into 1 cm $\times$ 1 cm square units for subsequent tests. The SEM images and size settings of different shaped structures are shown in Figure S1.


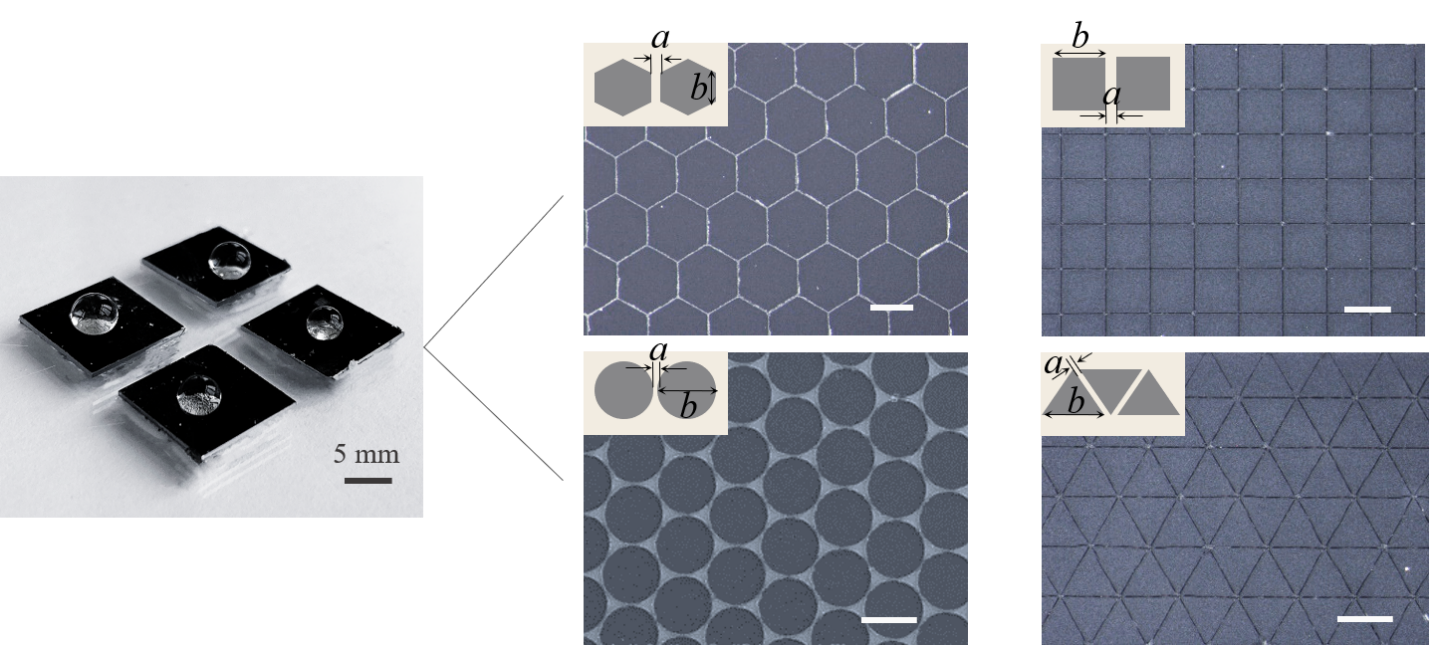


| **Figure S1.** The optical image of 1 cm × 1 cm pieces of the hierarchical patterned surfaces with the SEM images of the hierarchical surfaces containing variable patterns and the insets are corresponding size settings, where *a* = 5 μm and *b* = 50/100/150/200 μm (scale bars = 150 μm). |
| --- |

**S2. Transition from filmwise to dropwise condensation after VACNT planted**

The formation of discrete droplets and the corresponding condensation modes for a micropatterned substrate without implanted nanoarrays and the hierarchical surface are displayesd in Figure S2. Si surface is a hydrophilic material with an intrinsic contact angle of 75$^{\circ}$, and patterned Si surface can reach a contact angle of 121$^{\circ}$ after etching, suggesting the enhanced hydrophobicity on the Si surface due to the air pockets trapped in the etched cavities (see Figure S2(a)). However, the patterned Si surface is still unavailable for dropwise condensation. Figure S2(b) demonstrates the patterned Si surface which shows the undesired filmwise condensation. The hierarchical surface exhibits dropwise condensation after planting VACNT in the patterned grooves, as depicted in Figure S2(c). The transition of the hierarchical surface from filmwise to dropwise condensation proves that the nanoscale structure with high water-repellency exhibits an indispensable role to form dropwise condensation, whereas the patterned Si surface shows filmwise condensation even with the improved hydrophobicity.


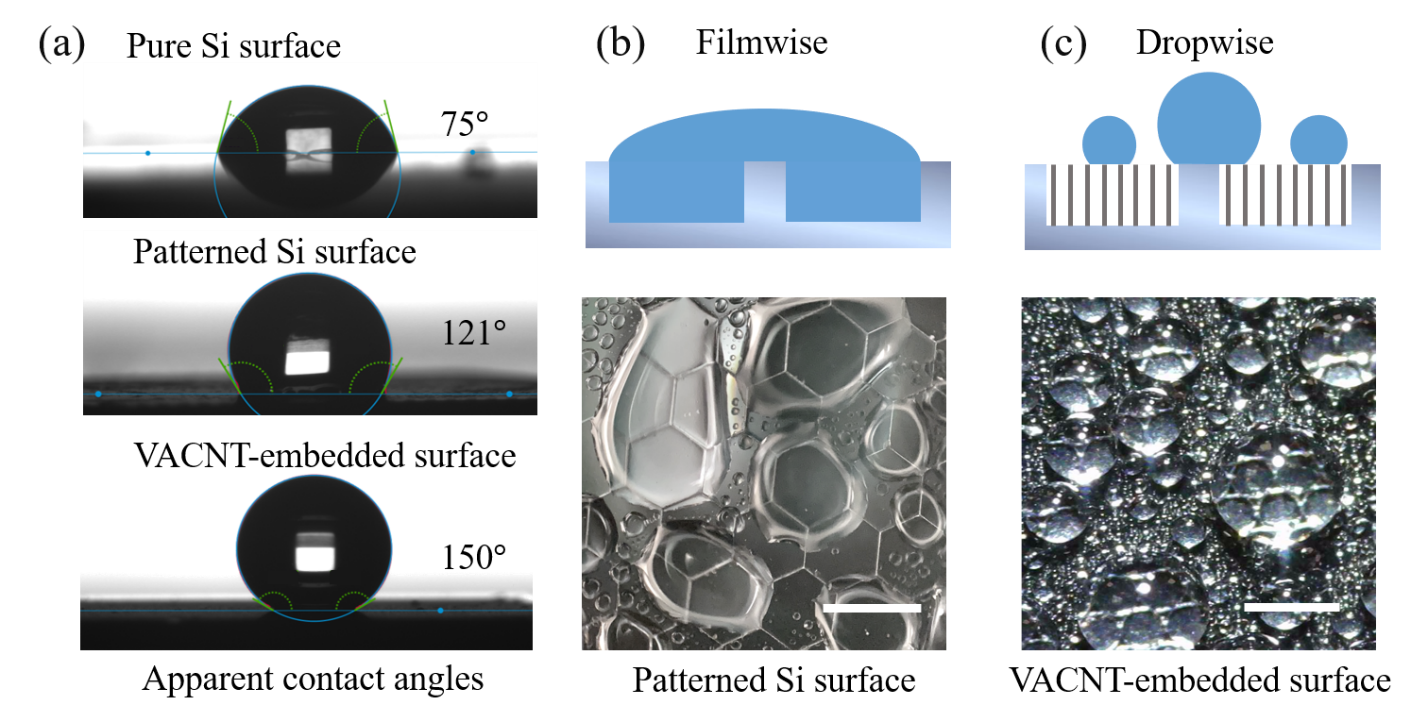


**Figure S2.** (a) Contact angles of the pure Si surface, the patterned Si surface and the VACNT-Si surface with the pattern length of 150 μm which varies from 75° to 150°. The optical image of (b) the filmwise condensation on the patterned Si surfaces, and (c) the dropwise condensation on the hierarchical VACNT-Si surface (scale bars = 400 μm) with the illustrations showing the corresponding condensation mode.

**S3.** **Thermal diffusivity of the hierarchical surfaces**

Figure S3 depicts the thermal diffusivity of the patterned Si surface and the hierarchical VACNT-Si surfaces with the same pattern length of 150 μm measured by a laser thermal conductivity meter at 𝑇 = 298 K. The thermal diffusivity increases from 38 mm^2^/s for a patterned Si surface to a maximum value of 87 mm^2^/s and an average value of 75 mm^2^/s for a hierarchical surface. Among the different pattern shapes, the circular-patterned surface has the largest proportion of Si area of 0.15, thus showing the least increase on the thermal diffusivity. In general, the average increase on the thermal diffusivity after VACNT implanted reaches 97%, which endows the hierarchical surfaces with an enhanced condensation property.

**
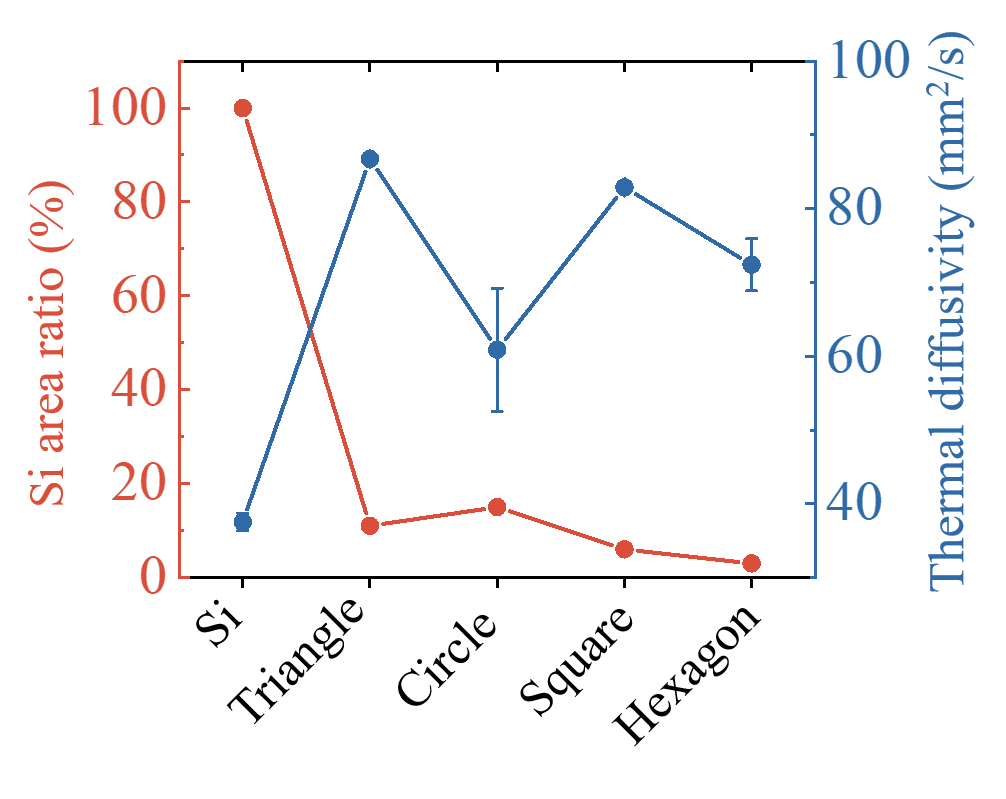
**

**Figure S3.** Thermal diffusivity and Si area ratio of the hierarchical surfaces and the pure-patterned Si surface for a length of 150 μm at 𝑇 = 298 K.

**S4.** **Contact area fraction measurement on the VACNTs**

Image contrast approach [1] is applied to determine the contact area fraction of the VACNTs planted on the hierarchical surfaces. A different height of CNTs exhibits variable brightness in the SEM image as shown in Figure S4(a). It is assumed that areas with a brightness higher than a certain threshold are considered as the top contact area exposed to vapor. The recognition process is carried out by the image processing software ImageJ and the contact area fraction is around 0.15 as demonstrated in Figure S4(b), while the surface contact fraction is within a range of 0.14~0.49 according to Refs. [1, 2].

**
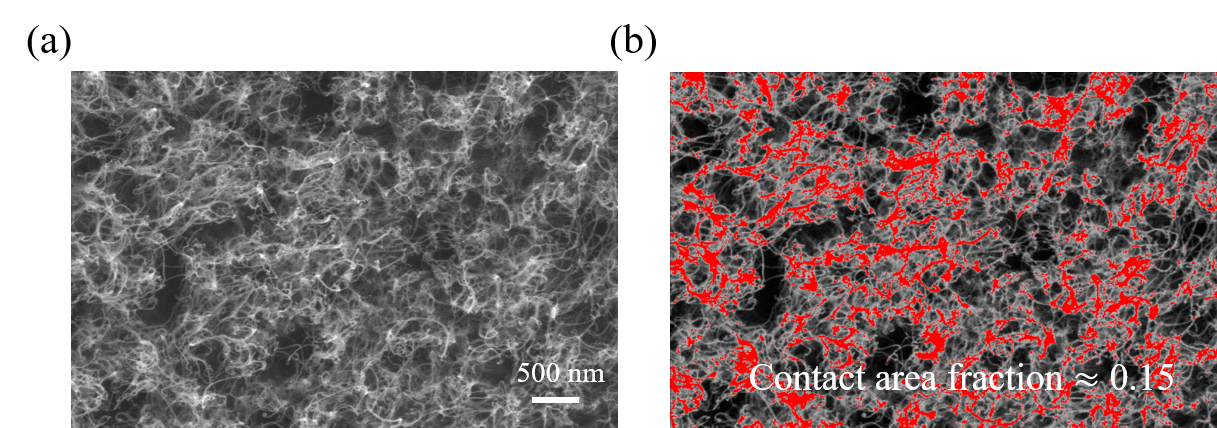
**

**Figure S4.** (a) SEM image of the VACNT on the hierarchical surfaces. (b) The recognition image after selecting bright pixels with the red area representing the top surface exposed to vapor.

**S5. Condensation model on heat transfer rate**

The condensation heat transfer model of one single droplet within the Cassie mode is illustrated in Figure S5(a). The temperature drop through a droplet can be expressed as

$\Delta T_{\mathrm{subcool}}=\Delta T_{i}+\Delta T_{\mathrm{curv}}+\Delta T_{\mathrm{drop}}+\Delta T_{\mathrm{nano}},$ (S1)

where $\Delta T_{i}$, $\Delta T_{\mathrm{curv}}$, $\Delta T_{\mathrm{drop}}$, $\Delta T_{\mathrm{nano}}$ are the temperature drops by the interfacial, the condensed drop curvature, the conduction through a condensed drop, and a rough nano-surface with pin structure, respectively. These four terms in S1 can be written as

$\Delta T_{i}=\frac{q}{h_{i}\left( 2\pi r^{2}\left( 1-cos\theta\right) \right)},$ (S2)

$\Delta T_{\mathrm{curv}}=\frac{r_{\min}}{r}\Delta T_{subcool,}$ (S3)

$\Delta T_{\mathrm{drop}}=\frac{q\theta}{4\pi rk_{c}sin\theta},$ (S4)

$\Delta T_{\mathrm{nano}}=\frac{qh}{k_{e}\pi\left( rsin\theta\right)^{2}},$ (S5)

where *q* is the heat transfer rate, $h_{i}$ is the interfacial heat transfer coefficient, $k_{c}$ is the thermal conductivity of condensed droplet, $r_{\min}=\frac{2\sigma_{c}T_{\mathrm{sat}}}{h_{\mathrm{fg}}\rho_{c}\Delta T_{\mathrm{subcool}}}$ is the minimum drop radius, and the effective thermal conductivity $k_{e}=f_{1}k_{s}+\left( 1-f_{1} \right)k_{a}$ considers the conductivities of solid-liquid contact fraction and the liquid-gas contact fraction which occurs at the gaps between the pins, in which $k_{s}$ is the thermal conductivity of the solid, and $k_{a}$ is the thermal conductivity of air for Cassie mode. Therefore, the heat transfer rate of a single droplet with a radius$r$ can be expressed as

$q\left( r \right)=\frac{\Delta T_{\mathrm{subcool}}\pi r^{2}\left( 1-\frac{r_{\min}}{r} \right)}{\left( \frac{h}{k_{e}{sin}^{2}\theta}+\frac{r\theta}{4k_{c}sin\theta}+\frac{1}{{2h}_{i}\left( 1-cos\theta\right)} \right)}.$ (S6)

According to the droplet population model, the heat transfer performance of the entire surface is quantified by applying population density theory for small drops $n\left( r \right)$ and the experimental-based equation for large drops $N\left( r \right)$ [3], that are

$N\left( r \right)=\frac{1}{3\pi r^{2}r_{\max}}\left( \frac{r}{r_{\max}} \right)^{-2/3},$ (S7)

$n\left( r \right)=\frac{1}{3\pi r_{e}^{3}r_{\max}}\left( \frac{r_{e}}{r_{\max}} \right)^{-2/3}\frac{r\left( r_{e}-r_{\min} \right)}{r-r_{\min}}\frac{A_{2}r+A_{3}}{A_{2}r_{e}+A_{3}}exp\left( B_{1}+B_{2} \right),$ (S8)

where $r_{e}$ is the effective radius, and $r_{\max}=\left( \frac{3}{sin\alpha}\frac{\left( cos\theta_{r}-cos\theta_{a} \right)sin\theta}{\left( 2-3cos\theta+{cos}^{3}\theta\right)}\frac{\sigma}{\rho g} \right)^{1/2}$ is the maximum drop radius. The parameters $A_{1}$, $A_{2},A_{3}$, $B_{1}$, and $B_{2}$ are given the same in Refs. [4, 5]. As depicted in the population distribution of drops with the different surface roughness in Figure S5(b), the number of droplets with a small size increases when the solid-liquid contact area decreases. The total heat flux of the dropwise condensation with Cassie wetting mode on a nanoscale roughness surface can be obtained as

$q_{\mathrm{total}}^{''}=\int_{r_{\min}}^{r_{e}} q\left( r \right)n\left( r \right)dr+\int_{r_{e}}^{r_{\max}} q\left( r \right)N\left( r \right)dr.$ (S9)


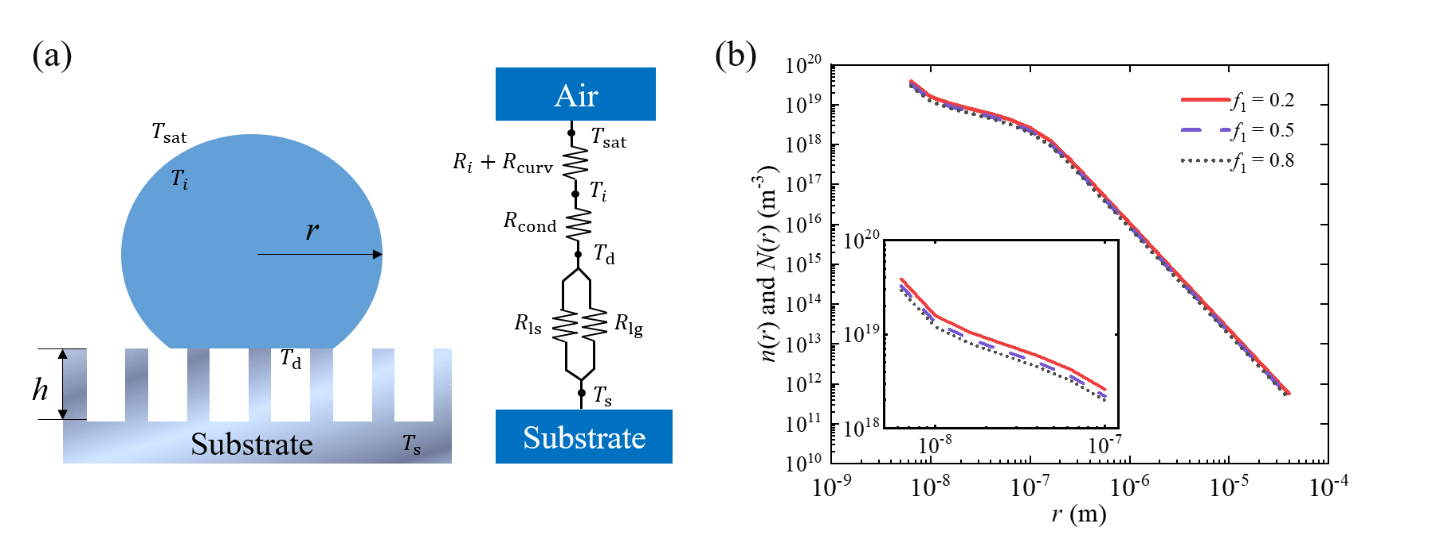


**Figure S5.** (a) Schematic heat transfer model and the resistance network on the rough surface with Cassie wetting state. (b) The number of condensed drops with respect to radius under the effects of surface roughness factor $f_{1}$.

**S6. Initial stage of condensation on the hierarchical surfaces**

Figure S6 shows the initial condensation behavior at 10^th^ s on the hierarchical surfaces as the pattern size *L* increases from 50 μm, 100 μm, 150 μm to 200 μm. Since water molecules tend to nucleate in the hydrophilic region, the surface (*L* = 50 $\mu m$) with more hydrophilic regions (10%) provides larger nucleation sites for vapor. At the initial stage of condensation (10 s), tiny droplets condense on the hydrophilic silicon wall even if the width is as narrow as 5 μm. We compute the number of nucleation sites at 10^th^ s through the coverage ratio of condensed droplets by image contrast approach. As shown in Figure S7, the hierarchical surface with *L* = 50 μm forms the largest surface coverage ratio of condensed droplets (70.4%), suggesting denser nucleation sites.

Figure S8 shows the condensation process of the hierarchical surfaces with different pattern sizes of 0 s, 15 s, and 30 s. Since water molecules tend to nucleate in the hydrophilic region, the surface with more hydrophilic regions (10%) condenses larger droplets (*L* = 50 μm). In contrast, the droplet size distribution is more uniform if a 97% area of VACNT is exposed (*L* = 200 μm). Although the combination of the hydrophilic region significantly enhances the ability to capture water molecules, the three-phase contact lines of droplets display no indication to pin on the edge of the patterns. This phenomenon proves that the hierarchical surfaces maintain an excellent hydrophobicity, and the hydrophilic regions enhance the overall condensation efficiency without reducing droplet mobility.

**
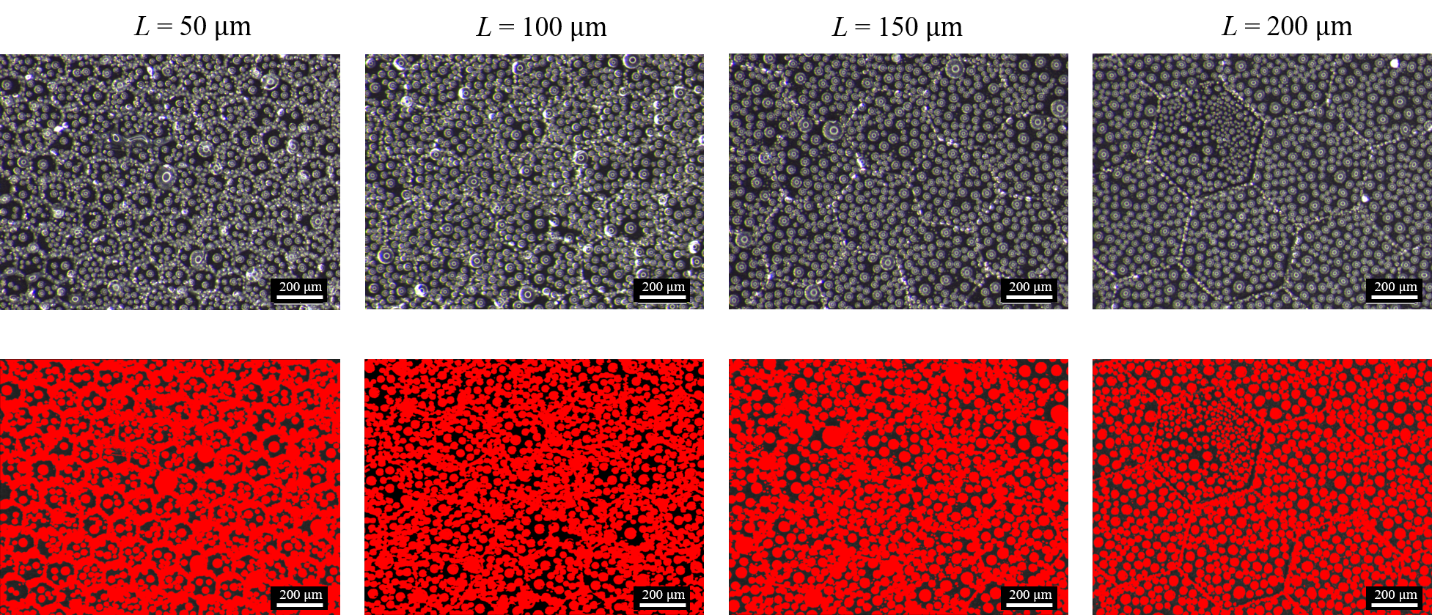
**

**Figure S6.** Optical images of the condensation performance at the 10^th^ s on the hierarchical surfaces as the pattern size *L* increases from 50 μm, 100 μm, 150 μm to 200 μm. ∆T_sub_ = 28 K. Corresponding recognition images after selecting bright pixels with the red area show the nucleation sites and distributions of condensed droplet.


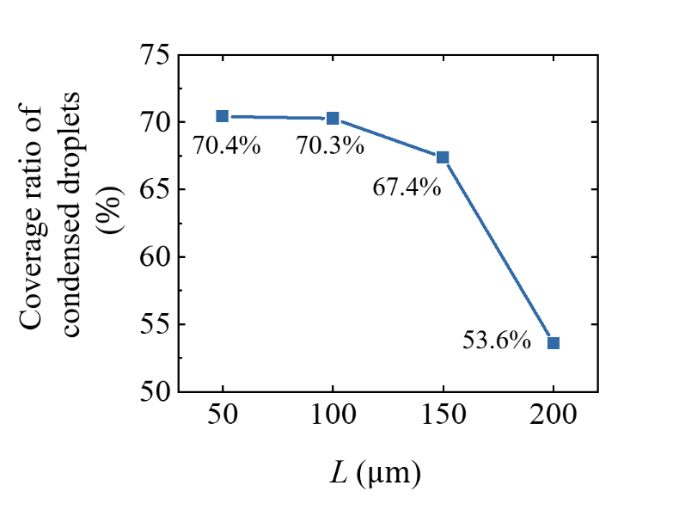


**Figure S7.** The coverage ratio of condensed droplets on the hierarchical surfaces as the pattern size *L* increases from 50 μm, 100 μm, 150 μm to 200 μm.

**
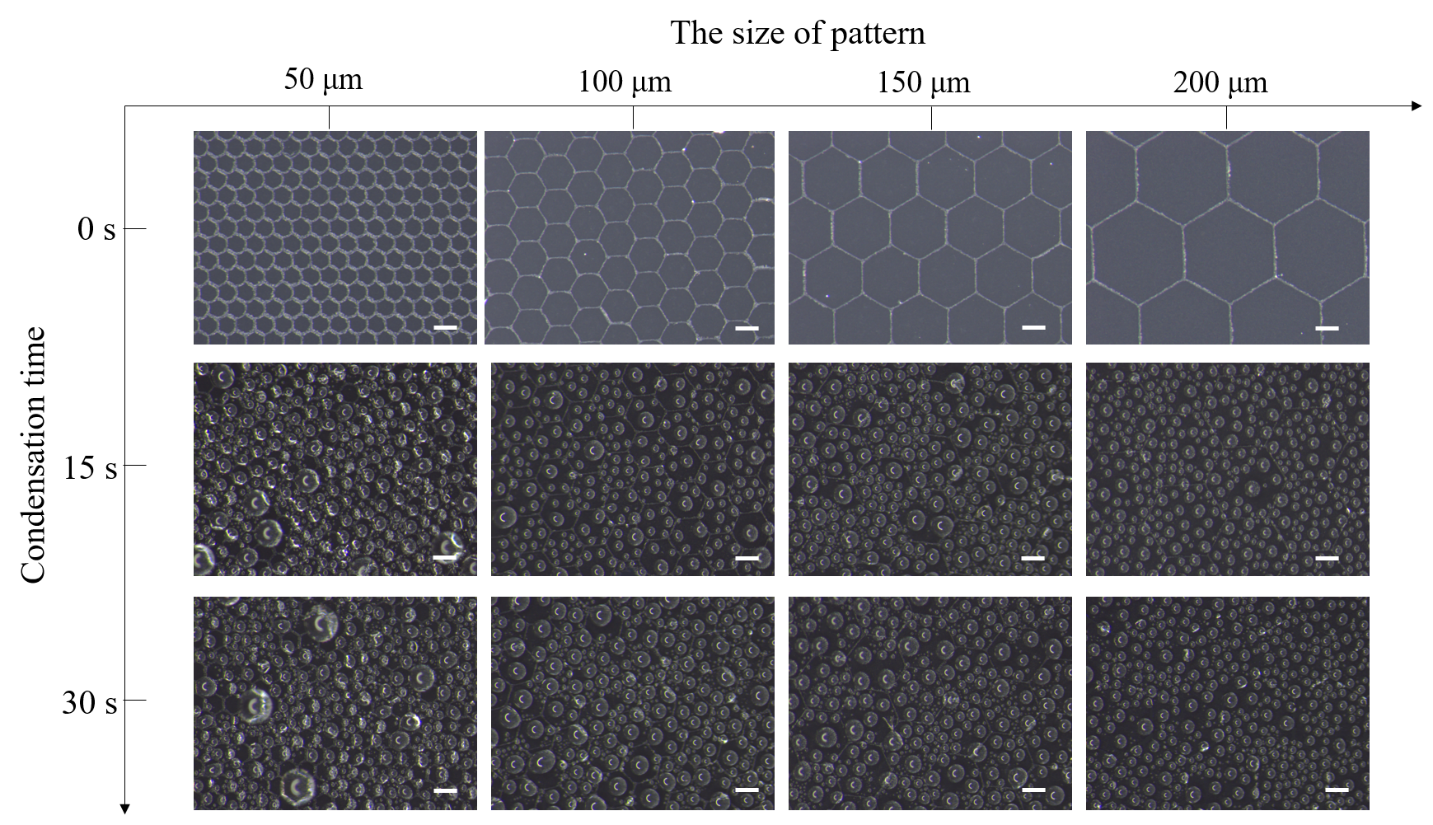
**

**Figure S8.** Optical images of condensation on the hierarchical surfaces with pattern sizes of 50, 100, 150, and 200 $\mu m$ after the condensation for 0, 15, and 30 seconds, respectively. Scale bars = 100 $\mu m$.

**S7. Size distribution of the condensation droplets on different surfaces**

Figure S9(a) demonstrates a violin plot for visualizing the distribution of condensed droplet diameter. The results show that the hierarchical surface with *L* = 50 μm has the widest range of droplet sizes than other cases. With the increase of *L*, the most droplet diameter tends to lie within the 20~80 μm range, which indicates the altofrequent condensed droplet sizes on the VACNT surface. Figure S9(b) shows the averaged size distribution of condensation droplets on surfaces with different *L* by repeating the test three times. When *L* equals to 50 μm, droplets can reach a size larger than 120 μm. This distribution suggests that the hierarchical surface with *L* = 50 μm prefers to condense larger droplets while leaving droplets with diameters less than 30 μm to adapt the small cells.

| **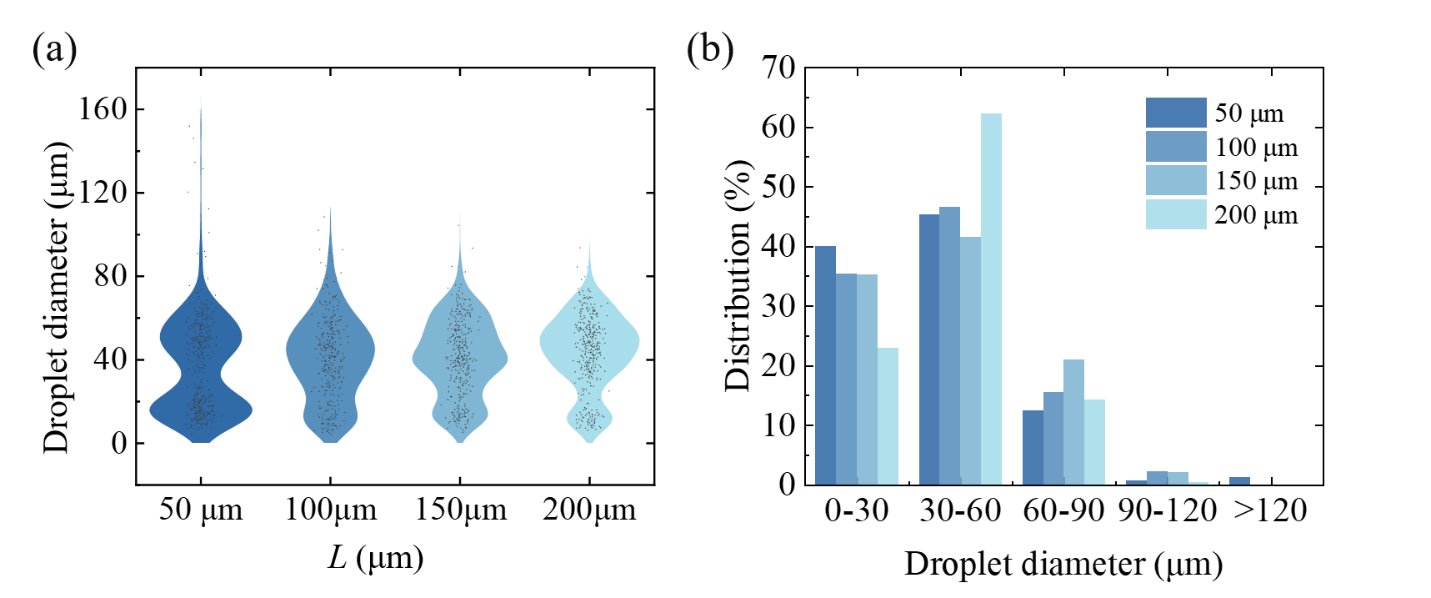** |
| --- |
| **Figure S9.** (a) The violin plot of the distribution of condensed droplet diameter. (b) The size distribution of condensation droplets on the hierarchical surface with different lengths of microstructure *L*. |

**S8.** **Size-dependent analyses on the condensation efficiency**

To enhance the condensation efficiency, the surface desires to have a large condensed droplet volume and a small coverage area under the droplets. To demonstrate a large size droplet having a higher efficiency than a small size droplet, we carry out the size-dependent analyses through the geometrical characteristic as shown in Figure S10. The spherical crown volume of the droplets denoted as $V={\pi R^{3}\left( 1-\cos\theta^{*} \right)}^{2}\left( 2+\cos\theta^{*} \right)/3$, and the $A_{\mathrm{proj}}=\pi R^{2}$ represents the maximum projection area of the droplet, where $R$ is the radius of the droplet and $\theta^{*}$ is the apparent contact angle. When a large-size droplet has the same $A_{\mathrm{proj}}$ with *n* number of small-size droplets ($n\geq2$), we obtained $R/r=\sqrt{n}$ ($r$ is the radius of the small-size droplet), and therefore derive ${V_{\mathrm{large}}}/{V_{\mathrm{small}}}={n^{\frac{3}{2}}}/n>1$ due to the equal $\theta^{*}$. Another case is that when a large-size droplet has the same $V$ with *n* number of small-size droplets, it can be calculated that $R/r=\sqrt[3]{n}$ and ${A_{\mathrm{proj}}^{\mathrm{large}}}/{A_{\mathrm{proj}}^{\mathrm{small}}={n^{\frac{2}{3}}}/n<1}$. The results show that the large-size droplets have a better performance on the condensation efficiency in terms of the balance between the condensed droplet volume and corresponding coverage area.

**
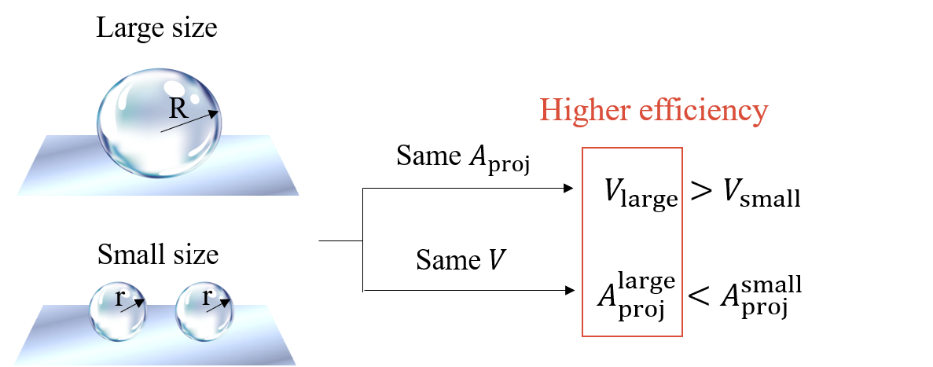
**

**Figure S10.** Size-dependent analyses on the condensation efficiency towards two conditions in terms of the balance between the condensed droplet volume and corresponding coverage area.

**S9.** **Performance of condensed droplets on the heterogeneous surface**

Figure S11(a) illustrates the molecular models used in the MD simulations. On the heterogeneous surfaces exposure to the hydrophilic region, water molecules tend to nucleate intensively in the hydrophilic region. The hydrophilic region becomes the nucleation center of condensation when it appears (Figure S11(b)), but the condensation nucleation sites are numerous and scattered on the hydrophobic VACNT (Figure S11(c)). The results show that the condensation evolution of droplets is related to the surface chemical properties, which demonstrates the water molecules face difficulties to nucleate on the hydrophobic VACNT surface, while the integration of the hydrophilic region plays a promising role in promoting condensation on the surface.


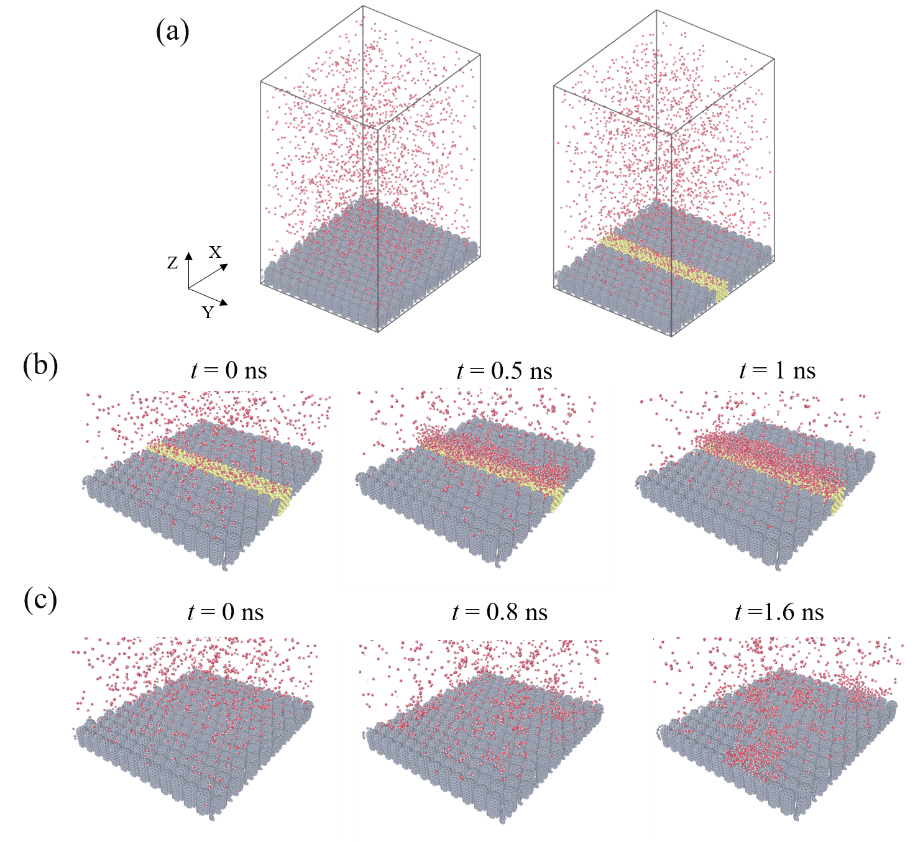


**Figure S11.** The water molecules prefer to accumulate in hydrophilic regions when condensing on heterogeneous surfaces. (a) The molecular models used in the MD simulations. Snapshots of the MD simulations during the condensation process on (b) the heterogeneous hierarchical surface, and (c) the VACNT surface.

**References**

[1] L. He, A. Karumuri, and S. M. Mukhopadhyay, "Wettability tailoring of nanotube carpets: morphology-chemistry synergy for hydrophobic-hydrophilic cycling," *Rsc Advances*, vol. 7, no. 41, pp. 25265-25275, 2017.

[2] Z. Ebrahim Nataj, A. S. Kazemi, and Y. Abdi, "Surface effects and wettability measurement considerations in fluorinated carbon nanotubes," *Applied Physics A*, vol. 127, no. 11, article 874, 2021.

[3] S. Kim, and K. J. Kim, "Dropwise condensation modeling suitable for superhydrophobic surfaces," *Journal of Heat Transfer-Transactions of the Asme*, vol. 133, no. 8, article 081502, 2011.

[4] S. Lee, H. K. Yoon, K. J. Kim et al., "A dropwise condensation model using a nano-scale, pin structured surface," *International Journal of Heat and Mass Transfer*, vol. 60, pp. 664-671, 2013.

[5] B. El Fil, G. Kini, and S. Garimella, "A review of dropwise condensation: Theory, modeling, experiments, and applications," *International Journal of Heat and Mass Transfer*, vol. 160, article 120172, 2020.
